# Lung ultrasound imaging in avian influenza A (H7N9) respiratory failure

**DOI:** 10.1186/2036-7902-6-6

**Published:** 2014-05-20

**Authors:** Nga Wing Tsai, Chun Wai Ngai, Ka Leung Mok, James W Tsung

**Affiliations:** 1 Adult Intensive Care Unit, Queen Mary Hospital, 102 Pok Fu Lam Road, Hong Kong, SAR China; 2 Accident and Emergency Department, Ruttonjee Hospital, 266 Queen’s Way, Wan Chai, Hong Kong, SAR China; 3 Department of Emergency Medicine, Division of Emergency Ultrasound, Mount Sinai School of Medicine, New York, NY 10029 USA

**Keywords:** Ultrasonography, Lung ultrasound, Influenza A virus, H7N9, Viral pneumonia, Point-of-care, Pandemics, Respiratory failure, Emergency medicine, Critical care medicine

## Abstract

**Background:**

Lung ultrasound has been shown to identify in real-time, various pathologies of the lung such as pneumonia, viral pneumonia, and acute respiratory distress syndrome (ARDS). Lung ultrasound maybe a first-line alternative to chest X-ray and CT scan in critically ill patients with respiratory failure. We describe the use of lung ultrasound imaging and findings in two cases of severe respiratory failure from avian influenza A (H7N9) infection.

**Methods:**

Serial lung ultrasound images and video from two cases of H7N9 respiratory failure requiring mechanical ventilation and extracorporeal membrane oxygenation in a tertiary care intensive care unit were analyzed for characteristic lung ultrasound findings described previously for respiratory failure and infection. These findings were followed serially, correlated with clinical course and chest X-ray.

**Results:**

In both patients, characteristic lung ultrasound findings have been observed as previously described in viral pulmonary infections: subpleural consolidations associated or not with local pleural effusion. In addition, numerous, confluent, or coalescing B-lines leading to ‘white lung’ with corresponding pleural line thickening are associated with ARDS. Extension or reduction of lesions observed with ultrasound was also correlated respectively with clinical worsening or improvement. Coexisting consolidated pneumonia with sonographic air bronchograms was noted in one patient who did not survive.

**Conclusions:**

Clinicians with access to point-of-care ultrasonography may use these findings as an alternative to chest X-ray or CT scan. Lung ultrasound imaging may assist in the efficient allocation of intensive care for patients with respiratory failure from viral pulmonary infections, especially in resource scarce settings or situations such as future respiratory virus outbreaks or pandemics.

**Electronic supplementary material:**

The online version of this article (doi:10.1186/2036-7902-6-6) contains supplementary material, which is available to authorized users.

## Background

The first human infections with avian influenza A (H7N9) associated with poultry exposure were reported in China in March of 2013[1]. Although mild illnesses have been observed, more concerning are the presentations of severe respiratory failure that have occurred in most cases, of which approximately one-third have resulted in death. No evidence of human-to-human transmission has been found so far[2]. However, a pandemic outbreak of this virus or other similarly lethal viruses such as MERS coronavirus or influenza A (H5N1) with rapid human-to-human spread would constitute a dire public health emergency.

Experiences with prior respiratory virus outbreaks[3–5] have demonstrated that point-of-care lung ultrasound can assist in distinguishing between various acute respiratory pathologies such as pneumonia[6], viral pneumonia[3–5, 7], and acute respiratory distress syndrome[8–10]. Point-of-care lung ultrasound may be a first-line diagnostic imaging alternative to chest X-ray early in the course of disease or CT scan in critically ill patients that cannot be moved[11] and may be used repeatedly to monitor disease progression[7] or resolution[12] especially in pandemic conditions when time and resources are scarce or overwhelmed[4].

## Methods

Our objectives are to describe lung ultrasound findings in two patients with severe respiratory failure from avian influenza A (H7N9) infection, provide clinical pathologic correlation, and to discuss the implications for viral pandemic preparedness.

## Results

### Case 1

**Figure 1:**
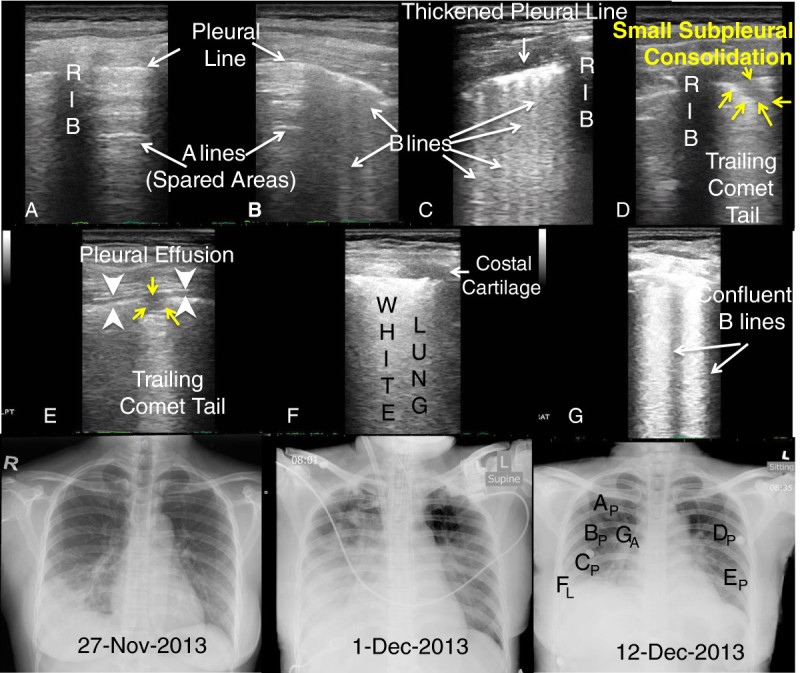
**LUS and CXR correlation from illness day (rows 1 & 2).** Panels **A-F**. **(A)** A lines = normal aerated (spared) lung; **(B & C)** B lines = interstitial lung water or pulmonary edema; **(D & E)** Small subpleural consolidation (yellow arrows); **(E)** Micro - Pleural Effusion (white arrow heads); **F**: White lung = ARDS, and **G**: Confluent B lines. Panel letters correspond to position letter on CXR (subscript P for posterior chest wall and subscript L for lateral chest wall LUS interrogation). Row 3: serial chest X-rays.

A 36-year-old Indonesian domestic worker who worked in Hong Kong for 10 years presented to Emergency Department in late November of 2013 for 4-day history of fever and cough. She traveled to Shenzhen, China, 10 days prior to her admission and visited a live poultry market with poultry contact. On presentation, she was febrile to 40°C, and the chest radiograph showed right lower lobe pneumonia (Figure 1, CXR from 27-Nov-2013)). She was initially admitted to the medical ward (day 1), but condition rapidly deteriorated requiring ICU admission and intubation on day 3. Right-sided pigtail catheter was inserted for right exudative pleural effusion. She was treated as community-acquired pneumonia. She developed acute respiratory distress syndrome, and ventilation became difficult despite high level of ventilatory support and prone position. Extracorporeal membrane oxygenation was started, and she was retrieved to a local center with extracorporeal membrane oxygenation support (ECMO) for further care on day 4.

Avian-origin influenza A (H7N9) virus was confirmed from nasopharyngeal aspirate, throat swab, and pleural fluid by real time reverse transcriptase-polymerase chain reaction. Treatment with intravenous zanamivir 600 mg twice daily was started. Empiric tazobactam/piperacillin and levofloxacin was also given. Her condition improved and she was extubated, and ECMO catheter was removed on day 10. H7N9 virus was found negative from day 9 onwards. A total 7-day course of intravenous zanamivir was completed, followed by prolonged course of oral oseltamivir. Patient was discharged from the intensive care unit on day 18 and was transferred to convalescent hospital for a course of pulmonary rehabilitation 2 weeks later. She was discharged home on day 45.

### Case 2

**Figure 2:**
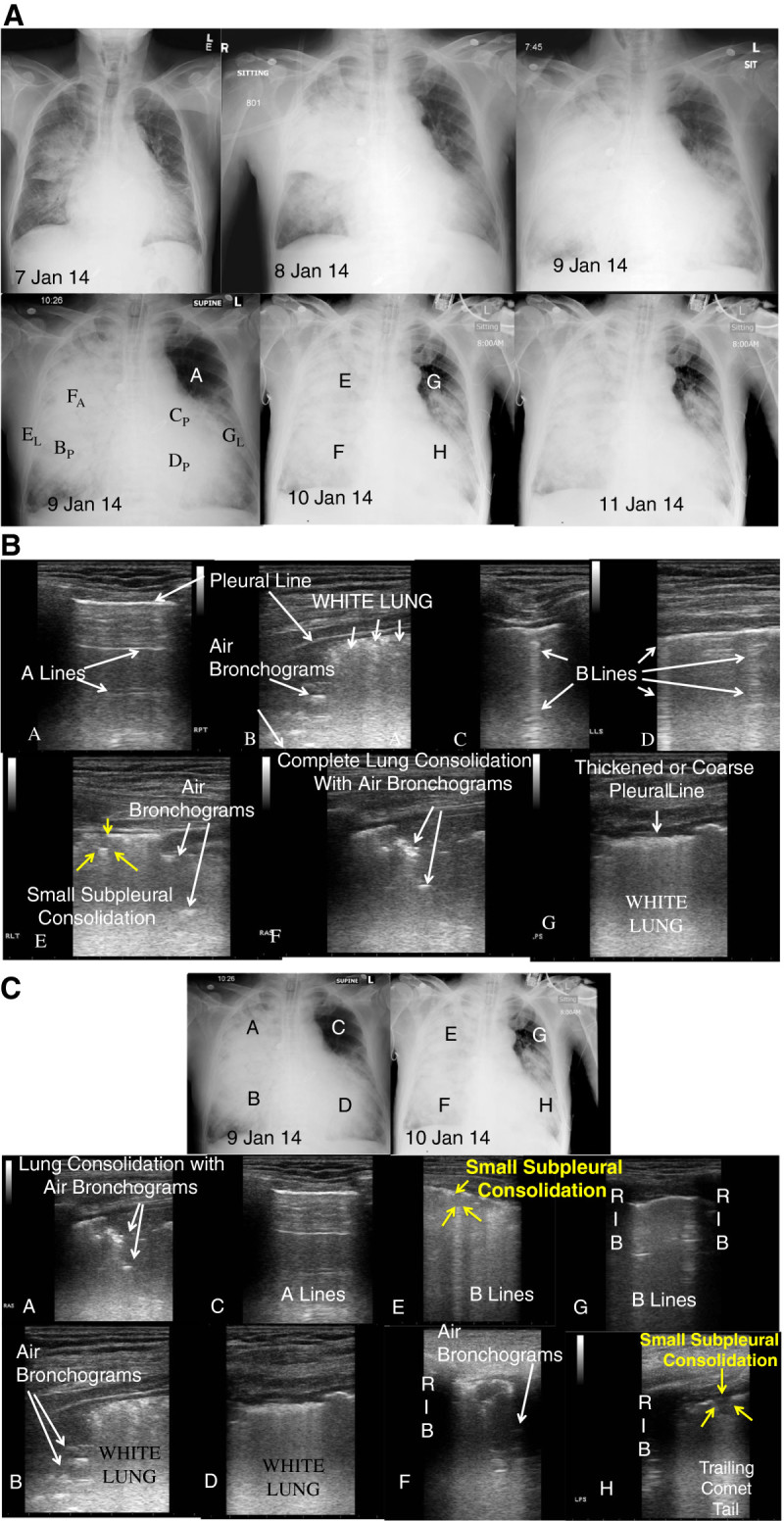
**Radiography and ultrasonography for case 2. (A)** Chest X-rays showing progression over course of illness**.** CXR Letters correspond to panel letters in Figure 2B and2C. Subscript A - anterior, P - posterior, and L- lateral. **(B)** Lung ultrasound images correlated to chest X-ray 9 Jan 14 in Fig.2A. Panels **A-G**. **(C)** Correlated lung ultrasound images with chest X-rays over 2 days showing disease progression, particularly the left upper lobe (panels **C** and **G**). Paired panels by anatomic area: A + E; B + F; C + G; and D + H.

**Figure 3:**
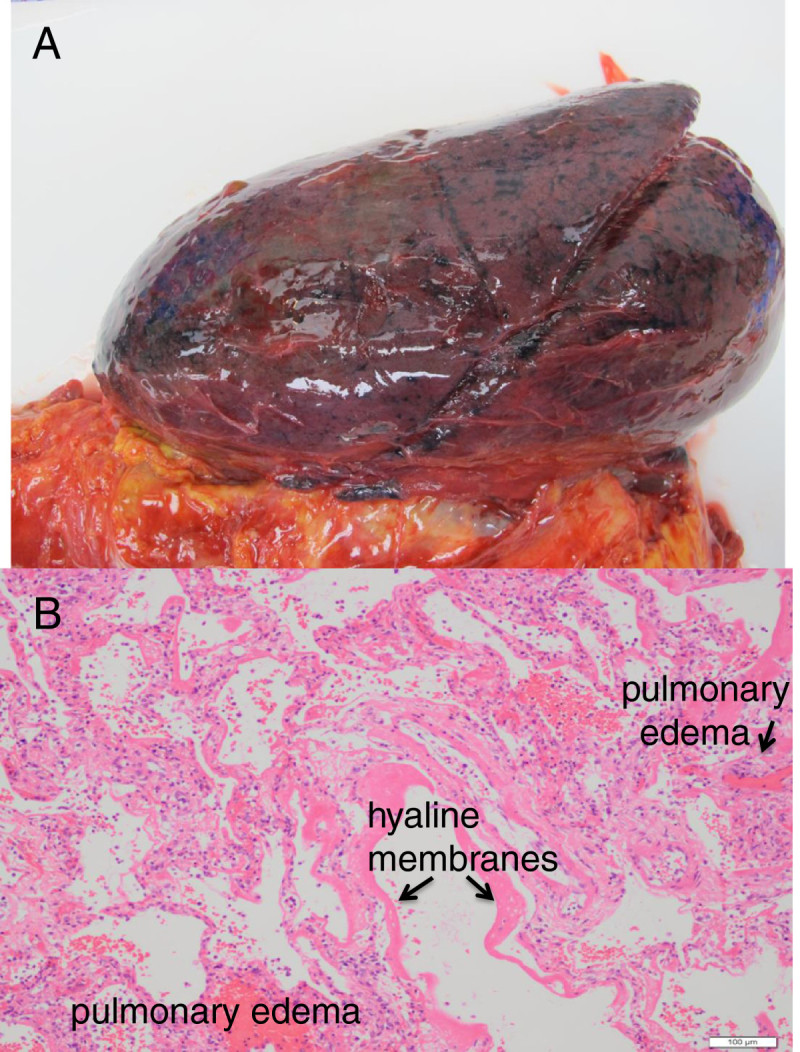
**Fresh gross anatomic pathology of left lung with pulmonary hemorrhage and edema (A).** Microscopic pathology showing diffuse alveolar damage, pulmonary edema, and hyaline membranes **(B)**.

A 65-year-old man with multiple medical conditions including renal failure and history of cardiothoracic surgery presented with fever, cough, and shortness of breath since 3 January. He was admitted to a local hospital in Hong Kong for chest infection 4 days later (Figure 2, CXR from 7 Jan 2014)). His condition rapidly deteriorated requiring intensive care unit admission on the same day and invasive mechanical ventilation and ECMO on the subsequent day. Further travel history revealed that the patient traveled to Shenzhen for 1 day just prior to his symptom onset. He passed by a wet market in Shenzhen but denied any direct poultry contact.Avian-origin influenza A (H7N9) virus was confirmed from nasopharyngeal aspirate and blood. IV zanamivir was started, and viral load was decreasing trend on daily monitoring. Empiric coverage with antibacterial including tazobactam/piperacillin and levofloxacin was given. No positive bacterial culture was grown from sputum and endotracheal aspirate to date. He developed acute cardiac arrhythmia, multiorgan failure with rapid deterioration and succumbed 1 week after hospital admission. Postmortem examination of the lungs (Figure 3) and heart was performed. Cardiomegaly was noted with no gross evidence of myocardial infarction of the heart. Pulmonary congestion and hemorrhagic changes were noted in bilateral lungs.

### Ultrasound technique, image analysis, and findings

Serial lung ultrasound (Esaote MyLabFive) with an 11- to 3-MHz linear array transducer was performed on day 16, day 21, and day 24 of case 1's hospital stay. They were performed on hospital days 3 and 4 for case 2. A six-zone scanning protocol[4] was used to image the lungs in perpendicular planes (transverse and coronal or parasagittal) for the anterior, lateral, and posterior lung area "AT the" midclavicular line, midaxillary line and parasaggital line medial to the scapulae. A modified protocol scanning along the midclavicular line, anterior-axillary, and posterior-axillary lines described by Lichtenstein and Meziere was used in case 2 due to critical illness[8]. Lung ultrasound examinations were compared and correlated to the chest radiograph done that day for both patients (Figures 1 and2).

Lung ultrasonographic findings included: (1) B-lines, defined as hyperechoic comet-tail artifacts extending from the pleural line to the bottom of the ultrasound screen which may be discrete (Figure 1B,C) or confluent (Figure 1F, Figure 3B,G), (2) lung consolidation with sonographic air bronchograms (Figure 2B,E,F, Figure 3A,B,F), (3) small subpleural consolidations (Figure 1D,E; Figure 3E,H), and (4) pleural effusion (Figure 1E, Figure 2C; Figure 3H). Interstitial syndrome was defined as the presence of three or more B-lines or if B-lines are confluent in an area or field of view or coalescing to become ‘white lung’ (Figure 3B,D). They may increase in number or in thickness to become confluent (Additional file1: Video S1; Figure 1B,C,F; Figure 3E) related to the increased amount of extravascular (interstitial) lung water[12]. Lung consolidations are echo-poor hypoechoic areas (hepatization) with air bronchograms that are depicted as hyperechoic linear elements (Additional file2: Video S2; Figure 2, Panels B, E and F)[6, 13]. Small subpleural consolidations that have been observed to be specific for viral infection[4, 7] are typically 0.25 to 0.5 cm in depth, contain no sonographic air bronchograms, associated with an interruption in the pleural line, and typically have a trailing comet tail artifact (Additional file3: Video S3)[4]. Pleural effusion is visualized on ultrasonography as an anechoic dependent collection bordered by diaphragm or visceral and parietal pleura.Additional file 1: **Video S1.** Series of video clips depicting progression of A-lines to B-lines, to confluent B-lines, to white lung (ARDS). (MPEG )Additional file 2: **Video S2.** Series of video clips demonstrating sub-pleural consolidations observed in viral pulmonary infections. (MPEG )Additional file 3: **Video S3.** Video clip of lung consolidation with sonographic air bronchograms consistent with pneumonia from patient 2. (MPEG )

## Discussion

The development and description of point-of-care lung ultrasonography has been reviewed, and evidence-based recommendations for its use published[11, 14]. Discrete ultrasonographic findings seen in our patients (B lines, confluent B lines, pleural effusion, and most specifically, small subpleural consolidations) have similarly been observed in other viral pulmonary infections such as measles, other influenza A subtypes (H1N1), and respiratory syncytial virus by multiple investigators around the world in pandemic and non-pandemic situations[3–5, 7]. Our second patient had co-existing consolidated pneumonia based on the finding of lung consolidation and air bronchograms visualized by ultrasound[6, 13].

These ultrasonographic findings can be investigated using a high frequency linear transducer as small subpleural consolidations as well as small pleural effusions can be missed with lower frequency curvilinear probes and cannot be visualized by chest X-ray[4, 15, 16]. Larger footprint lower frequency curvilinear (up to 60 mm in length) or microconvex probes can be used to rapidly assess the extent of lung pathology, especially in patients with impending respiratory failure[5, 15]. Interstitial syndrome on ultrasound is visualized as numerous B-lines (at least 3 per field of view)[8, 11, 12]. Acute respiratory distress syndrome (ARDS) is seen as the predominant presence of confluent B lines ( at least >3 B lines per field) or white lung associated with pleural line abnormalities described as thickening (>2 mm) or coarsening, with few spared areas (observation of A lines)[10]. Bacterial (consolidated) pneumonia is distinguished from viral (interstitial) pneumonia by ultrasonography visualized as lung consolidations with sonographic air bronchograms, typically larger than 0.5 cm in depth[4, 5, 7, 16, 17].

Our cases were confirmed to have influenza A H7N9 by RT-PCR, where prior research efforts to characterize the diagnostic accuracy of these findings have been hampered by the lack of access to an appropriate or logistically feasible reference gold standard for viral pneumonia[4, 5, 7]. However, it has been noted that these findings have very high interobserver reliability (Cohen's *K* = 0.82)[4] and can be detected early in the course of disease when chest X-ray may often be normal[5]. Additionally, numbers of viral ultrasound findings have been shown to correlate with disease severity in admitted infants with viral bronchiolitis, with resolution of ultrasound findings as symptoms resolve[7].

This high interobserver agreement in ultrasonographic findings may promote reduced practice variation in antibiotic or antiviral medication prescribing relative to chest X-ray for improved antibiotic or antiviral medication stewardship. In severe cases, point-of-care lung ultrasound may lead to efficient allocation of resources such as respiratory isolation rooms, ventilatory support, and ECMO. It is unclear if different virus types (e.g., H7N9 vs. H5N1 vs. H1N1 vs. RSV) manifest different patterns in viral lung ultrasonographic findings (relative numbers of B lines, confluent B lines, and small subpleural consolidations). Further investigation into these ultrasound patterns may allow distinguishing between different virus types on the basis of ultrasonographic findings[18]. Autopsies of patients with influenza A (H5N1) virus infection have ‘shown diffuse alveolar damage with hyaline membrane formation, patchy interstitial lymphoplasmacytic infiltrates, bronchiolitis with squamous metaplasia and pulmonary congestion with varying degrees of hemorrhage’[19]. Our second patient had similar findings on postmortem examination (Figure 3). We speculate that these interstitial infiltrates and pulmonary congestion would appear on ultrasound as B lines or confluent B lines (or white lung), with hemorrhage appearing as small subpleural consolidations similar to that observed in our cases and in other viruses.

From a practical and logistical viewpoint of managing large numbers of patients during a pandemic outbreak, point-of-care lung ultrasonographic evaluation can be performed more rapidly, efficiently, and cheaply than chest X-ray. During pandemic overcrowding from 2009 influenza A (H1N1), emergency department volumes quadrupled and waiting times for chest X-ray tripled from a median of 29 to 98 min, contributing to delays for all patients requiring imaging[4]. With reported median lung ultrasound exam times of 6 min[4], point-of-care ultrasound can be used to reduce ED congestion and is scalable by increasing numbers of portable ultrasound units with clinicians capable of performing lung ultrasound. For patients too critically ill to be transported for CT scan, point-of-care ultrasound is a feasible imaging alternative[8]. A greater concern when evaluating children who are at risk for higher mortality from avian influenza A (H5N1) infection[19] is that ultrasonography avoids radiation exposure that elevates future cancer risk when using chest X-ray or CT scan[20, 21].

Our first patient had all of the sonographic findings (B lines, confluent B lines, small subpleural consolidations, spared areas, and pleural effusion) described for ARDS[10] that were noted to be resolving on serial ultrasonographic examinations with clinical improvement. Our second patient had similar findings with ominous changes in the left upper lobe with normal aeration (A lines) initially, progressing to interstitial syndrome (B lines) or pulmonary congestion (Figure 2C,G) noted on serial lung ultrasounds prior to succumbing. These findings are similar to ultrasound findings described with other influenza A subtypes (H1N1 and seasonal), as well as other viral pulmonary infections[3–5].

## Conclusions

Clinicians with access to point-of-care ultrasonography may use these findings as an alternative to chest X-ray or CT scan. Lung ultrasound imaging may help guide triage of resources[4] (e.g., respiratory isolation rooms, ventilators), medical decision-making[5] (e.g. antivirals, antibiotics, fluid administration, or ECMO) and monitor disease progression or resolution with therapy, especially in resource scarce settings or situations such as future respiratory virus outbreaks or pandemics[19, 22].

### Consent

Written informed consent was obtained from the patient (for case 1) and the patient's next of kin (for case 2) for publication of this report and any accompanying images.

## Electronic supplementary material

### Authors’ original submitted files for images

Below are the links to the authors’ original submitted files for images.Authors’ original file for figure 1Authors’ original file for figure 2Authors’ original file for figure 3Authors’ original file for figure 4Authors’ original file for figure 5
