# Supplementary figures and images for: Lung ultrasound imaging in avian influenza A (H7N9) respiratory failure
Source: Crit Ultrasound J. 2014 May 20;6:6. doi: 10.1186/2036-7902-6-6 (PMC4051407; doi:10.1186/2036-7902-6-6)

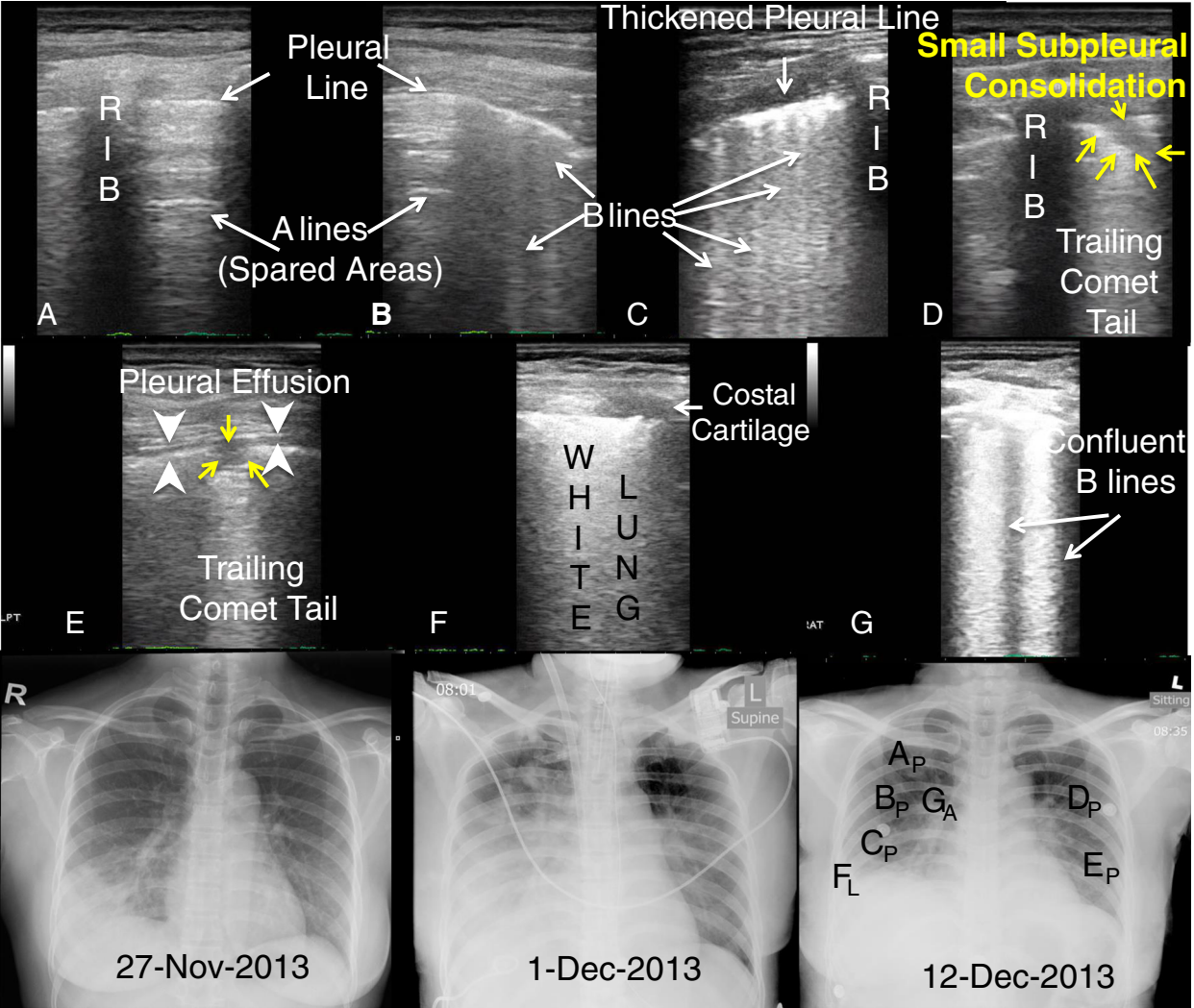

Supplement: Supplementary file 4 — Authors’ original file for figure 1 [file 13089_2014_108_MOESM4_ESM.pdf]

**A**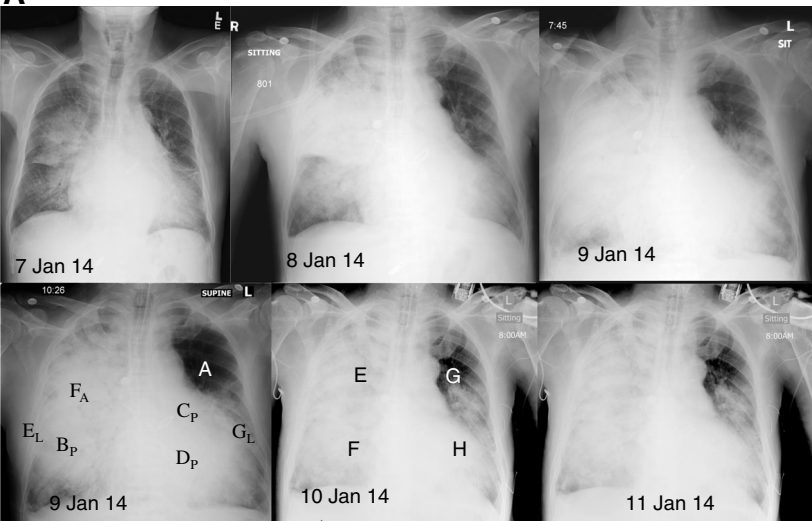**B**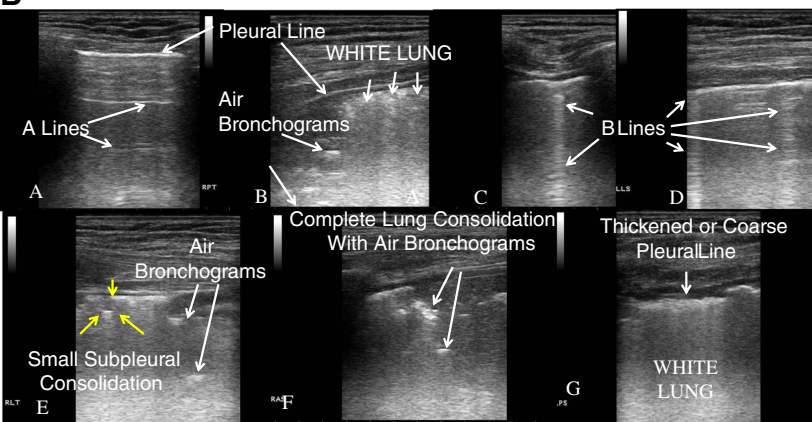**C**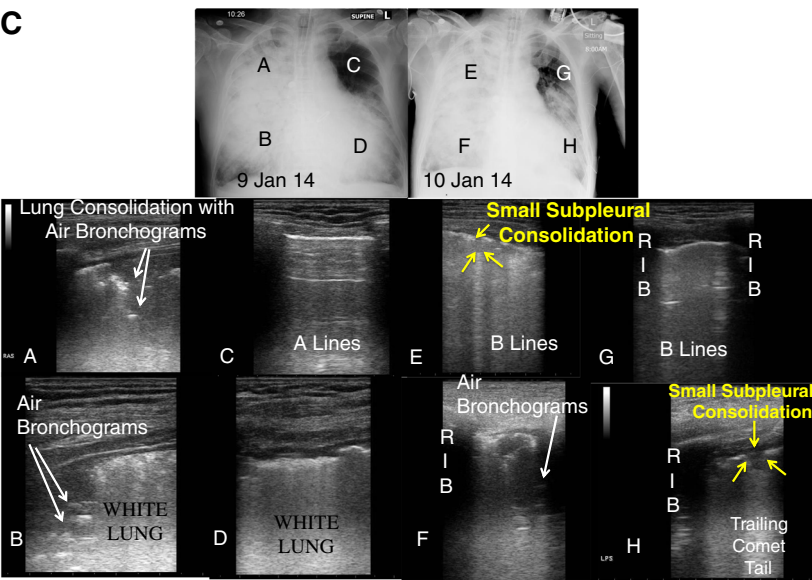

Supplement: Supplementary file 5 — Authors’ original file for figure 2 [file 13089_2014_108_MOESM5_ESM.pdf]

A

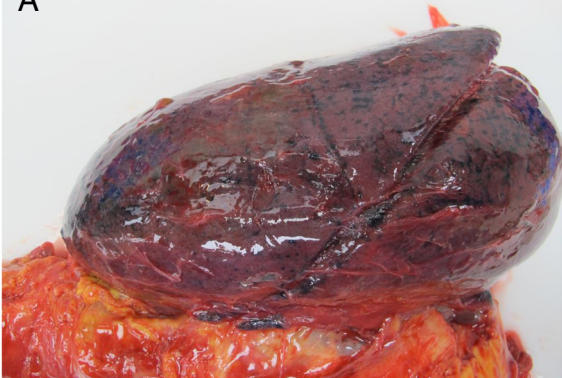

B

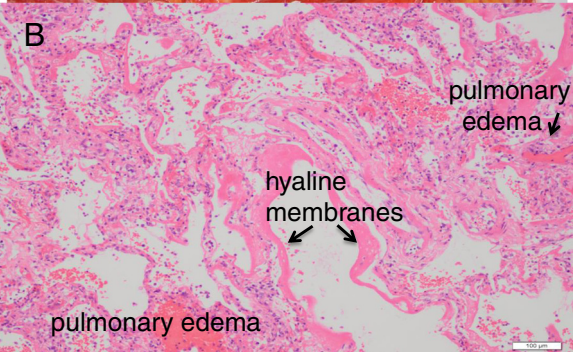

Supplement: Supplementary file 6 — Authors’ original file for figure 3 [file 13089_2014_108_MOESM6_ESM.pdf]

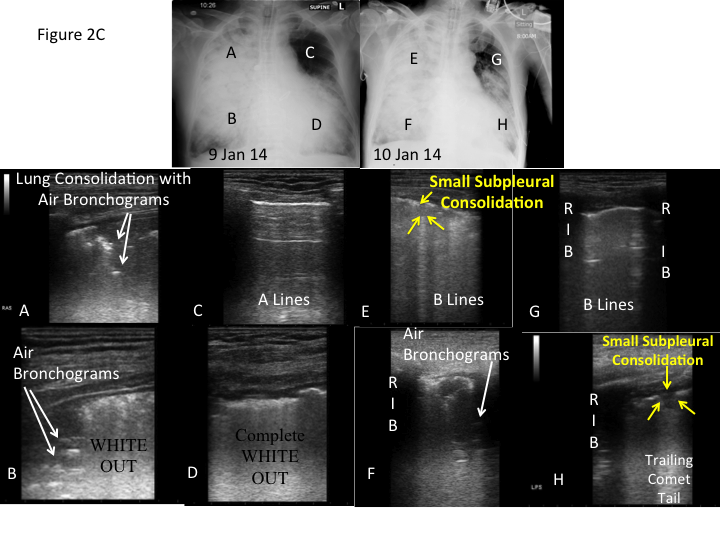

Supplement: Supplementary file 7 — Authors’ original file for figure 4 [file 13089_2014_108_MOESM7_ESM.tiff]

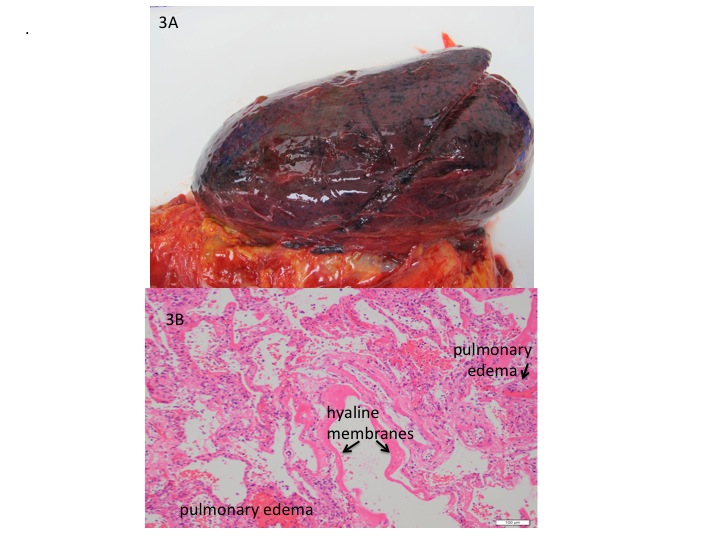

Supplement: Supplementary file 8 — Authors’ original file for figure 5 [file 13089_2014_108_MOESM8_ESM.jpeg]
